# Digital Health Applications (DiHA): Approaches to develop a reimbursement process for the statutory health insurance in Austria

**DOI:** 10.1016/j.hlpt.2023.100780

**Published:** 2023-09

**Authors:** Gregor Goetz, Reinhard Jeindl, Dimitra Panteli, Reinhard Busse, Claudia Wild

**Affiliations:** a HTA Austria - Austrian Institute for Health Technology Assessment GmbH, Vienna, Austria; b Department of Health Care Management, Berlin Institute of Technology, Berlin, Germany

**Keywords:** Digital health applications, mHealth, Health app, Reimbursement

## Abstract

**Purpose:**

To elaborate a concept for implementing digital health applications (DiHA), including prioritisation criteria (PC) for the Austrian context and an overview of available prioritised DiHAs.

**Methods:**

Based on European DiHA-listings and input by Austrian experts, a categorised meta-directory of DiHAs was created. PC were developed to reflect, inter alia, the provisions of the Austrian General Insurance Act, and were applied to the meta-directory to identify DiHAs potentially relevant for the Austrian statutory health insurance. An iterative process with expert involvement was used to tailor an existing reimbursement framework to the Austrian setting.

**Results:**

The meta-directory comprised 132 DiHAs. Developed PC focused on plausibility (German language) and legal aspects (treatment/monitoring of chronic conditions), while other criteria (e.g. interoperability standards) were considered optional. After applying the PC, 38 DiHAs were potentially relevant in the Austrian setting. Of these, only seven supported current health record integration. Most of the prioritised DiHAs reported on CE marking (29/38) and data protection (35/38), while reporting on risk class (10/38) and technical algorithms (0/38) was sparse. For DiHA reimbursement, a four-step process is proposed: identification (ideally based on needs assessment); filtering based on PC; review of technical, regulatory and evidentiary requirements; and health technology assessment.

**Conclusion:**

The proposed concept can offer guidance for policy makers (e.g., on prioritising available DiHAs) and may further foster scientific debate with regard to DiHA implementation. Further discussion on how to fully incorporate regulatory, technical, and evidentiary criteria is needed. Attention should be given to national implementation requirements, re-assessment criteria, and appropriate remuneration schemes.

## Highlights

• Currently, there is no universal approach for the categorisation of digital health applications.
• The high number of available digital health applications requires a filtering system for reimbursement decisions.
• The reimbursement approach depicted in this article, incorporating regulatory, technology-specific as well as evidence-based criteria, offers guidance for policy-makers.
• Certain national implementation requirements (such as compatibility with electronic health records) require further attention.

## Introduction

Digital health applications (DiHAs) can be defined as digital medical devices that can support the treatment of illnesses or the compensation of impairments. In addition to smartphone applications, they can include browser-based web applications or software for use on classic desktop computers [1]. The main function of the DiHA is based on digital technologies that support the detection, monitoring, treatment, compensation or mitigation of disease, injury or disability [2]. DiHAs can be seen as a component of electronic health (eHealth), with mobile devices (e.g., cell phones) monitoring, inter alia, vital signs of patients in a medical care context [3].

On a regulatory level, the European Union (EU) Medical Device Regulation 2017/745 (MDR), in force since May 2021, has brought noteworthy developments with regard to DiHAs [4]: medical software is regarded as a medical device if it is intended to fulfil medical purposes according to the manufacturer. As a result, the risk classes of many DiHAs have been upgraded to a higher level [5].

Internationally, the reimbursement and implementation of DiHAs are still in their infancy, with notable differences concerning the frameworks selected to evaluate what will be covered [6]. That is to say; some European countries have established transparent directories of available, evaluable and reimbursable DiHAs. In Germany, for instance, the Digital Care Act (Digitale Versorgungsgesetz - DVG, Dec. 2019) [7] laid the groundwork for prescribing of selected DiHAs by physicians and therapists that are reimbursed by the statutory health system. For this purpose, the Federal Institute for Drugs and Medical Devices (BfArM) created a list of reimbursable DiHA, the DiHA directory (*DiGA-Verzeichnis*). As of the beginning of August 2022, there are 35 reimbursable DiHAs in this directory [8]. Similarly, France, Belgium, and England have created directories of DiHAs, although their exact purpose and process for inclusion differ. Most countries have not developed a transparent reimbursement process for DiHA yet. In Austria, for instance, there is currently no established process for determining the reimbursement and implementation of DiHAs, although a process to establish a transparent reimbursement framework in the future has been initiated. However, DiHAs are already used within disease management programs (DMPs) for patients with chronic diseases [9,10].

Because of the numerous fields of potential DiHA adoption, generic benefit assessments are likely to be inadequate. Instead, the additional benefit of a DiHA is dependant on its function, the target group and the intended effect as proposed within a categorisation approach developed by a team of researchers from the TU Berlin [11]. Although high expectations with regard to the additional benefit of implementing DiHAs are present, there is a general lack of sound scientific evidence to support the use of most DiHAs. This is also due to the fact that the evaluation of DiHAs is complex (e.g., frequent software updates), requiring a comprehensive and tailored methodological approach [11].

In 2020, a systematic analysis was conducted by the Austrian Institute for Health Technology Assessment (AIHTA) [5,6]. Drawing on six international assessment frameworks, it proposed to use a tiered approach considering risk classes with subsequent evaluation of HTA aspects relevant in Austria. For DiHA meeting the requirements, the relevant HTA aspects can then be defined, based on manufacturers' claims of DiHA functionality and intended use, and evaluated as a result in order to make an informed decision on the proven benefits and expected effects of the DiHAs. Further specifics and options relating to an Austrian evidence-based reimbursement process, such as legal aspects, considerations from health insurers, as well as technical interoperability factors, were not explored further.

The purpose of this paper is to elaborate a concept for a reimbursement process of Digital Health Apps (DiHAs) for Austrian statutory health insurers. As part of this process, we aimed to develop a model to prioritise DiHAs that are directly relevant to the Austrian context based on technical knowledge (e.g., law, IT infrastructure, etc.). To provide an overview of prioritised DiHAs, we applied the model to DiHA lists from selected European DiHA directories.

## Methods

Building on a first draft for a process and evaluation of DiHAs in Austria derived from an overview of DiHA evaluation frameworks [6] and a categorisation approach from the TU Berlin [11], we developed a prioritisation model, summarised technical knowledge to be integrated to the model, and identified and summarised available DiHAs.

### Identification

**Fig. 1:**
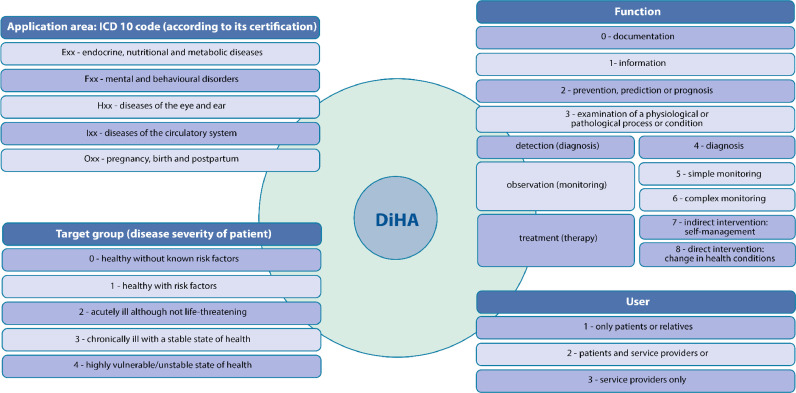
Categorisation approach based on the iDiGA-project (TU Berlin).

Source: Retrieved and slightly adapted from [11].

For the identification of potentially eligible DiHAs for Austria, a meta-directory of DiHAs was created. For this purpose, a focused search for DiHAs from listings and directories from European countries was conducted. We further carried out expert consultations to identify DiHAs developed or currently in use in Austria. We then categorised each identified DiHA from the meta-directory based on a recent classification framework [11] (see Fig. 1) and excluded DiHAs that are to be used solely by service providers on the system level, such as health system managers or data services. Then, we quantified the distribution of the DiHA target groups, functions, and indication areas, for DiHAs available in German language. Each step was carried out by one person (GG or RJ) and verified by another person (RJ or GG).

### Prioritisation based on legal requirement

For the prioritisation of potentially relevant DiHAs for the Austrian statutory health insurance, expert consultations were conducted: (i) to establish criteria for selecting relevant DiHAs that fall in the scope of Austrian health insurance, and (ii) to define technology-specific requirements when implementing DiHAs into the Austrian health system. For this, we presented the overview of the categorised DiHAs to experts and discussed eligibility for Austria based on, inter alia, legal and technical considerations.

### Technical and regulatory requirements

For providing an overview on prioritised DiHAs and potential technical characteristics, we first clustered the prioritised DiHAs according to ICD indication areas [12]. The reported technical interoperability standards were then compared to the current technical requirements of the Austrian electronic health record (ELGA) for an assessment of compatibility [13,14].

### Evidence requirements according to risk classification

Finally, the prioritised DiHAs were categorised according to their risk classes and the 2020 version of the National Institute for Health and Care Excellence (NICE) evidence standards framework (ESF) for digital health technologies [15] was used to determine function specific evidence requirements.

### Concept for systematic and evidence-based reimbursement process

To fine-tune conceptualising options for a systematic and evidence-based reimbursement process for Austria, we used an iterative process with expert involvement. This built on a previous concept for a multistage process suggested by AIHTA based on an analysis of internationally available assessment frameworks for DiHAs [5]. Expert consultations aimed at collecting technical knowledge [16] specific to the Austrian context (e.g., IT infrastructure, Austrian law). Experts were selected to capture perspectives relevant for the evaluation and implementation of DiHAs, as identified from a previous review of international frameworks [6].

Experts from the following institutions/ areas of expertise were consulted by means of face-to-face, telephone and video call interviews and/or e-mail exchange depending on their availability:

• A lawyer with expertise in the Austrian General Social Insurance Act (ASVG),
• CEO of the Austrian electronic health record (ELGA),
• CEO of the IT services of the social insurances (ITSV),
• Head of Department of Healthcare Provision and Innovation Management at Dachverband der Sozialversicherungsträger (DVSV) and
• CEO of Health Hub Vienna

## Results

### Identification: meta-directory of European DiHAs

**Fig. 2:**
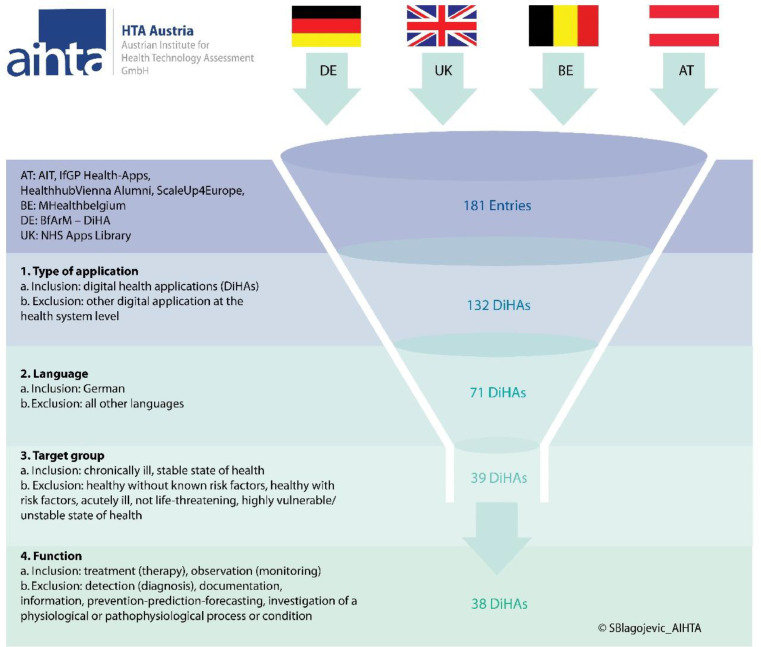
Prioritisation of digital health applications potentially relevant for Austria. **Abbreviations:** AIHTA – Austrian Institute for Health Technology Assessment; AT – Austria; BE – Belgium; BfArM - Bundesinstitut für Arzneimittel und Medizinprodukte; DE – Germany; DiHA(s) – digital health application(s); IfGP – Institut für Gesundheitsförderung und Prävention GmbH; NHS – National Health Service; UK – United Kingdom.

**Sources:** AT: Listings received by Austrian experts and partly available online (https://www.ifgp.at/cdscontent/load?contentid=10008.652551&version=1521703724; https://healthhubvienna.at/); BE: MHeallthbelgium (https://mhealthbelgium.be/); DE: BfArM – DiHA (https://diga.bfarm.de/de;); UK: NHS Apps Library (https://digital.nhs.uk/services/nhs-apps-library). All websites accessed in August 2021.

We identified seven directories/ listings of DiHAs from four countries (Belgium, Germany, United Kingdom and Austria). Directories from Belgium (mHealthBelgium), Germany (BfArM – DiHA) and the United Kingdom (NHS Apps Library) are accessible online. Austrian experts consulted for this work provided official listings of DiHAs from Austrian institutions (Austrian Insitute of Technology, Institut für Gesundheitsförderung und Prävention, Healthhub Vienna Alumni, ScaleUp4Europe) which are not fully accessible online. More details on the sources can be found within the legend of Fig. 2. Overall, the meta-directory contained 176 entries after de-duplication. Of these, 132 DiHAs met the definition of a DiHA used for this work. There were 71 DiHAs available in German language. Over half of these DiHAs (*n* = 39) target chronically ill people, and a substantial number of DiHAs (*n* = 25) aim at supporting healthy people with no known previous illness or risk factors. On the contrary, only a few of these DiHAs target healthy individuals with risk factors (*n* = 5) or acutely ill individuals (*n* = 2). Further, none of the identified DiHAs available in German language was designed to help vulnerable patients with unstable health conditions.

The distribution of DiHAs according to their function shows that a large proportion of them are in the field of monitoring or therapy (*n* = 41). Furthermore, there are DiHAs with informative (*n* = 22), diagnostic (*n* = 6) or documentary (*n* = 2) functions. Table 1 gives an overview of the distribution of DiHAs available in German by target group and function.

**Table 1:** Distribution of DiHAs available in German language according to function and target group.

**Function of the DiHA**	**0 - Healthy, without known risk factors**	**1 - Healthy, with risk factors**	**2 - Acutely ill, although not life-threatening**	**3 - Chronically ill with a stable state of health**	**Total**
0 - Documentation	2	–	–	–	2
1 - Information	17	2	2	1	22
4 - Diagnosis	4	2	–	–	6
5 - Simple monitoring	2	1	–	3	6
6 - Complex monitoring	–	–	–	9	9
7 - Indirect intervention (self-management)	–	–	–	23	23
8 - Direct intervention (change in health conditions)	–	–	–	3	3
**Total**	**25**	**5**	**2**	**39**	**71**

### Prioritisation: development and application of criteria for defining the relevant DiHA in scope with legal requirements

Based on expert consultations and on the General Social Insurance Act (ASVG) scope of reimbursable interventions [17], criteria have been developed to filter those DiHAs that may be directly relevant to the Austrian system and as such require an assessment. In so doing, the following criteria were developed:

• **Language:** DiHA is available in German language
• **Target group:** Chronically ill, with a stable health status
• **Function of the DiHA:** Treatment and monitoring

These prioritisation criteria have plausible (i.e., language) and legal (chronically ill concerning treatment/ monitoring) roots. For the latter, and according to the Austrian General Social Insurance Act (ASVG), medical treatment must be sufficient and expedient but must not exceed what is necessary. Through health care services, the ability to work and take care/ provide for one's own vital needs should be restored, strengthened, consolidated or improved as far as possible (§ 133 para. 2 ASVG) [18]. By Austrian law, a reimbursement decision must be based on the criteria of appropriateness (suitability for achieving the goal of health treatment), necessity (this also includes the level of care to be guaranteed by solidarity), effectiveness (proof through studies) and cost-effectiveness. DiHAs that exclusively include functions in the field of health promotion and prevention, for instance, do not constitute statutory health insurance benefits under the ASVG system. However, there is currently no precise legal regulation of the reimbursement of DiHAs in Austria (for more information, see [17] and pp. 33/34 in [10]).

Based on the expert consultations, further technical components were discussed: The Austrian ELGA currently only supports the CDA-7 standard. This potential technical aspect (interoperability standard) was deemed as highly relevant, but from the health insurer's perspective not suitable as a prioritisation criterion, because interoperability was judged as a dynamic field.

After having applied these additional prioritisation criteria relevant to the Austrian context to the 132 identified DiHAs, 38 DiHAs were available (see Fig. 2).

### Technical requirements for and characteristics of 38 DiHAs according to risk class and evidence levels

Based on former work from the AIHTA on assessment frameworks [5,6], the following variables were then defined and relevant data were extracted primarily from the DiHA manufacturer websites:

• DiHA name and manufacturer
• CE mark
• EU MDR 2017/745 risk class [4]
• Data protection statement
• Technical interoperability standard
• Information on used algorithms

The indication area with the most available DiHAs were mental disorders, with nine available DiHAs. There were seven DiHAs available to support the care of patients with cardiovascular diseases and a further seven for endocrine/ nutritional/ metabolic diseases. Further, there were some 15 “other” indications, covering some indications with fewer available DiHAs or DiHAs that may be covered in multiple indications.

For all 38 included DiHAs that met the prioritisation criteria, the highest evidence standards according to the NICE framework classification (C, interventions) is applicable. Information on the functionality of the underlying algorithms was not provided for any of the 38 DiHAs.

### Mental disorders

The indication area with the most available DiHAs was mental disorders. Nearly all (8/9) of these DiHAs had a CE mark and a privacy statement aligned with the General Data Protection Regulation (GDPR). The manufacturer of one of these DiHAs did not report on CE marking and is currently under re-assessment concerning data storage. None of the nine mental health DiHAs had reported a risk class (according to the MDR). For most of the DiHAs supporting the management of mental health disorders, information on interoperability standards was not available online. For the three DiHAs with available information, interoperability is currently not compatible with the Austrian ELGA (see Table 2).

**Table 2:** Digital health applications supporting the treatment of patients with mental disorders.

DiHA name (manufacturer)	Target group/ Function	CE mark *	2017/745 risk class*	Data protection⁎⁎	NICE evidence standards framework classification [15]	Technical interoperability standard	Compatibility with ELGA	Information on used algorithms	Source⁎⁎⁎
Catch It	chronically ill; indirect intervention	NR	NR	*Re*-assessment ongoing	C	NR	NR	NR	https://www.liverpool.ac.uk/csd/app-directory/catch-it/
Deprexis (GAIA AG)	chronically ill; indirect intervention	Yes	NR	Yes	C	HL7 FHIR	No	NR	https://info.deprexis.com/
Invirto (Sympatient GmbH)	chronically ill; direct intervention	Yes	NR	Yes	C	NR	NR	NR	https://invirto.de/
Kalmeda (mynoise GmbH)	chronically ill; direct intervention	Yes	NR	Yes	C	NR	NR	NR	https://www.kalmeda.de/
Mindable: Panikstörungen und Agoraphopie (Mindable Health GmbH)	chronically ill; direct intervention	Yes	NR	Yes	C	NR	NR	NR	https://www.mindable.health/
Selfapys Online-Kurs bei Depressionen (Selfapy GmbH)	chronically ill; indirect intervention	Yes	NR	Yes	C	NR	NR	NR	https://www.selfapy.com/kurse/depression
somnio (mementor DE GmbH)	chronically ill; indirect intervention	Yes	NR	Yes	C	NR	NR	NR	https://somn.io/
velibra (GAIA AG)	chronically ill; indirect intervention	Yes	NR	Yes	C	HL7 FHIR	No	NR	https://de.velibra.com/
vorvida (GAIA AG)	chronically ill; indirect intervention	Yes	NR	Yes	C	HL7 FHIR	No	NR	https://de.vorvida.com/

⁎ according to manufacturer website.

⁎⁎ data protection statement (compliance with the General Data Protection Regulation, including data storage in the EU) Abbreviations: CE - european conformity (“conformité européenne”); GDPR - general data protection regulation; MDR - medical device regulation; NR - not reported.

⁎⁎⁎ cited 30.09.2021.

### Cardiovascular diseases

amongst the seven DiHAs supporting the management of cardiovascular diseases (through complex monitoring or indirect interventions), four DiHAs were reported to have a CE mark, and six DiHAs reported a concordance with the European data regulation. For two DiHAs, a IIa risk class was reported. The interoperability standard was reported for only one of these DiHAs and was compatible with the Austrian ELGA (see Table 3).

**Table 3:** Digital health applications supporting the treatment of patients with cardiovascular diseases.

DiHA name (manufacturer)	Target group/ function	CE mark*	2017/745 risk class*	Data protection⁎⁎	NICE evidence standards framework classification [15]	Technical interoperability standard	Compatibility with ELGA	Information on used algorithms	Source⁎⁎⁎
CardiacSense (Arseus Hospital NV)	chronically ill; complex monitoring	Yes	2A	Yes	C	NR	NR	NR	https://www.cardiacsense.com/
CardioMemory	chronically ill; indirect intervention	NR	NR	Yes	C	NR	NR	NR	https://kit.ait.ac.at/wp-content/uploads/2018/06/TGD-Tirol-Steiermark-VAEB_V2.0–2018–03–30_AIT.pdf
FibriCheck (Qompium)	chronically ill; complex monitoring	Yes	2A	Yes	C	NR	NR	NR	https://www.fibricheck.com/
Heartfish	chronically ill; indirect intervention	NR	NR	Yes	C	NR	NR	NR	https://www.heartfish.io/
HerzMobil	chronically ill; indirect intervention	NR	NR	Yes	C	HL7 CDA	Yes	NR	https://kit.ait.ac.at/wp-content/uploads/2018/06/TGD-Tirol-Steiermark-VAEB_V2.0–2018–03–30_AIT.pdf
Rehappy (Reha GmbH)	chronically ill; indirect intervention	Yes	NR	Yes	C	NR	NR	NR	https://www.rehappy.de/
MobECG (Savvy)	chronically ill; complex monitoring	Yes	NR	NR	C	NR	NR	NR	http://www.savvy.si/en/

⁎ according to manufacturer website.

⁎⁎ data protection statement (compliance with the General Data Protection Regulation, including data storage in the EU) Abbreviations: CE - european conformity (“conformité européenne”); GDPR - general data protection regulation; MDR - medical device regulation; NR - not reported.

⁎⁎⁎ cited 30.09.2021.

### Endocrine, nutritional and metabolic diseases

For the indication area of endocrine, nutritional and metabolic diseases, seven DiHAs were available. Five DiHAs reported that a CE mark is available. Information on data storage was available for all seven DiHAs, with six being in line with the GDPR. Out of the seven DiHAs, risk class was reported as IIa for two DiHAs and IIb for one further DiHA. Interoperability standards were reported for three DiHAs, with two of these DiHAs compatible with the Austrian ELGA (see Table 4).

**Table 4:** Digital health applications supporting the treatment of patients with endocrine, nutritional and metabolic diseases.

DiHA name (manufacturer)	Target group/ function	CE mark*	2017/745 risk class*	Data protection⁎⁎	NICE evidence standards framework classification [15]	Technical interoperability standard	Compatibility with ELGA	Information on used algorithms	Source⁎⁎⁎
DiabCare	chronically ill; indirect intervention	NR	NR	Yes	C	NR	NR	NR	https://kit.ait.ac.at/wp-content/uploads/2018/06/TGD-Tirol-Steiermark-VAEB_V2.0–2018–03–30_AIT.pdf
DiabMemory 2	chronically ill; indirect intervention	NR	NR	Yes	C	NR	NR	NR	https://kit.ait.ac.at/wp-content/uploads/2018/06/TGD-Tirol-Steiermark-VAEB_V2.0–2018–03–30_AIT.pdf
Freestyle LibreLink – BE (Abbott SA/NV)	chronically ill; complex monitoring	Yes	2b	Yes	C	KMEHR, HL7 CDA, HL7 FHIR, SNOMED-CT	Yes	NR	https://myfreestyle.be/fr/
Guardian Connect App (Medtronic Belgium)	chronically ill; complex monitoring	Yes	2A	No‡	C	NR	NR	NR	https://guardianconnect.medtronic-diabetes.co.uk/
mySugr Tagebuch-App	chronically ill; indirect intervention	Yes	2A	Yes	C	KMEHR, HL7 CDA, HL7 FHIR, SNOMED-CT	Yes	NR	https://www.mysugr.com/en/diabetes-app/
Vitadio	chronically ill; indirect intervention	Yes	NR	Yes	C	NR	NR	NR	https://vitad.io/
zanadio (aidhere GmbH)	chronically ill; indirect intervention	Yes	NR	Yes	C	HL7 FHIR	No	NR	https://zanadio.de/

⁎ according to manufacturer website.

⁎⁎ data protection statement (compliance with the General Data Protection Regulation, including data storage in the EU) Abbreviations: CE - european conformity (“conformité européenne”); GDPR - general data protection regulation; MDR - medical device regulation; NR - not reported.

⁎⁎⁎ cited 30.09.2021.

‡ Privacy policy according to the GDPR available, storage location of data outside the EU.

### Other indications

Further, fifteen DiHAs out of the 38 are to be used for other indications (e.g. specific neurological, orthopaedic or oncological indications) or general/multiple purposes (e.g. for "patients with chronic diseases/ infectious diseases/ patients with long-term medication"). For twelve of these DiHAs, a CE mark was reported. Fourteen DiHAs reported on data storage, with thirteen DiHAs aligned with the data protection regulation of the EU and one further DiHA with data stored outside of the EU. For five out of fifteen DiHAs, a risk class was reported: three reported risk class I, and two reported risk class IIa. For seven DiHAs, the interoperability standards were reported, of which four use an interoperability standard that is compatible with the Austrian ELGA (see Table 5).

**Table 5:** Digital health applications supporting the treatment of specific other indications or to be used for multiple purposes.

DiHA name (manufacturer)	Target group/ Function	CE mark*	2017/745 risk class*	Data protection⁎⁎	NICE evidence standards framework classification [15]	Technical interoperability standard	Compatibility with ELGA	Information on used algorithms	Source⁎⁎⁎
Vivira (Vivira Health Lab GmbH)	chronically ill; indirect intervention	Yes	NR	Yes	C	NR	NR	NR	https://www.vivira.com/
M-sense Migräne (Newsenselab GmbH)	chronically ill; indirect intervention	Yes	NR	Yes	C	NR	NR	NR	https://www.m-sense.de/
AirviewTM (Resmed)	chronically ill; indirect intervention	Yes	2A	Yes	C	KMEHR, HL7 CDA, HL7 FHIR, SNOMED-CT	Yes	NR	https://www.resmed.com/en-us/healthcare-professional/products-and-support/monitoring-and-data-management/airview/
CANKADO PRO-React Onco (CANKADO Service GmbH)	chronically ill; indirect intervention	Yes	NR	Yes	C	GAMP-5, ICH GCP E6(R2) HL7 FHIR	No	NR	https://diga.cankado.com/
Comarch Home Health (Comarch)	chronically ill; complex monitoring	Yes	2A	NR	C	NR	NR	NR	https://www.comarch.com/healthcare/products/comarch-homehealth/
Comunicare (Comunicare Solutions)	chronically ill; complex monitoring	Yes	1	Yes	C	KMEHR, HL7 CDA, HL7 FHIR, SNOMED-CT,	Yes	NR	https://www.comunicare.be/home/
Elevida (GAIA AG)	chronically ill; indirect intervention	Yes	NR	Yes	C	HL7 FHIR	No	NR	https://elevida.de/
Florio (Swedish Orphan Biovitrum (Belgium) BV)	chronically ill; simple monitoring	Yes	1	Yes	C	NR	NR	NR	https://florio-haemo.com/
HumanITcare	chronically ill; complex monitoring	NR	NR	Yes	C	NR	NR	NR	https://humanitcare.com/
icompanion (icometrix)	chronically ill; simple monitoring	Yes	NR	No1	C	NR	NR	NR	https://icompanion.ms/
Mika (Fosanis GmbH)	chronically ill; indirect intervention	Yes	NR	Yes	C	HL7 FHIR	No	NR	https://www.mitmika.de/
Mindmate	chronically ill; indirect intervention	NR	NR	Yes	C	NR	NR	NR	https://www.mindmate-app.com/
Noona (Varian Medical Systems Belgium)	chronically ill; simple monitoring	Yes	NR	Yes	C	KMEHR, HL7 CDA, HL7 FHIR, SNOMED-CT	Yes	NR	https://www.varian.com/products/software/care-management/noona
Plateforme Maela (Medtronic Belgium)	chronically ill; complex monitoring	Yes	1	Yes	C	KMEHR, HL7 CDA, HL7 FHIR, SNOMED-CT	Yes	NR	https://www.maela.fr/de/
Vaica	chronically ill; indirect intervention	NR	NR	Yes	C	NR	NR	NR	https://www.vaica.com/

⁎ according to manufacturer website.

⁎⁎ data protection statement (compliance with the General Data Protection Regulation, including data storage in the EU) Abbreviations: CE - european conformity (“conformité européenne”); GDPR - general data protection regulation; MDR - medical device regulation; NR - not reported.

⁎⁎⁎ cited 30.09.2021.

## Concept for an evidence-based reimbursement process of DiHAs in Austria

**Fig. 3:**
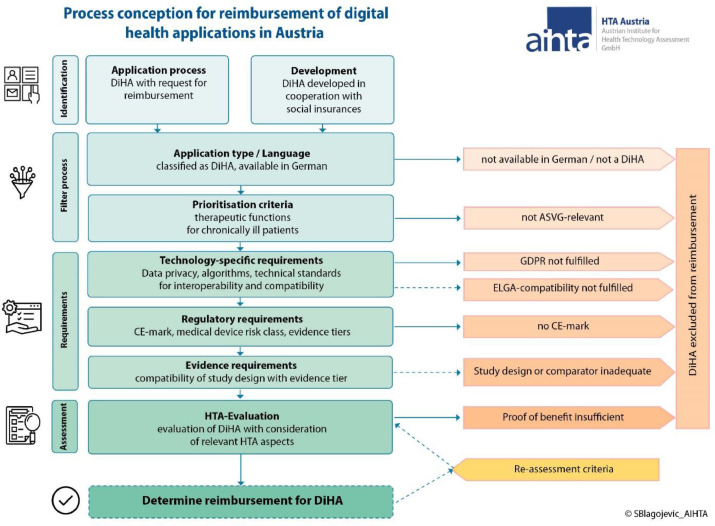
Process conception for the reimbursement of digital health applications in Austria. **Abbreviations:** ASVG – Austrian General Social Insurance Act; CE – Conformitè Europëenne; DiHA – digital health application; ELGA – Austrian electronic health record; GDPR – General Data Protection Regulation; HTA – health technology assessment.

Fig. 3 shows a concept for an evidence-based reimbursement process for DiHAs in Austria based on previous work by AIHTA [5,6] and building on further insights of the previously described meta-directory and the prioritisation criteria to be found above. It also visualises some decisions to be taken by policy makers. The concept consists of four steps before a DiHA can be reimbursed:

**Identification.** Relevant DiHAs can be co-designed within the identification phase based on a previous "need-assessment" (in cooperation with the social insurance). Additionally, an application procedure for manufacturers may be developed. In this stage, information on the DiHA may be provided (e.g., in the form of an application dossier).

**Filtering and prioritisation.** Available DiHAs may be first categorised according to the categorisation scheme (see Fig. 1). Then, the developed prioritisation criteria may apply: For a DiHA to be relevant in the statutory health insurance for Austria, German language availability is a requirement. Additionally, therapeutic functions for chronically ill patients are the prioritised criteria for a DiHA to be considered relevant, in accordance with the ASVG.

**Technical, regulatory and evidence requirements**. Within this phase, regulatory (CE mark, risk class, evidence level) and technology-specific (data privacy, interoperability, algorithms) requirements are reviewed. We further propose to use the NICE evidence framework to determine evidence requirements for the specific DiHAs [15]. When the next version of the NICE evidence standards framework is released (to be published August 9th, 2022), the updated version should be used [15]. DiHAs that don't fulfil the GDPR requirements are proposed to be excluded from reimbursement, while compatibility with ELGA is recommended but may not strictly be required.

**Benefit assessment**. After a DiHA has fulfilled the requirements of the previous phases, an evidence synthesis may be conducted to determine the benefit provided by the DiHA (e.g., medical, social or organisational) in line with established HTA methodology. For this, as recommended in the previous work by AIHTA [5], the HTA module for mobile medical applications [19] as well as the Digi-HTA framework [20] may be used. Certain criteria for a re-assessment (e.g., due to continuous software updates) would still be necessary.

A reimbursement of DiHAs may then be considered, although the remuneration model would still need to be discussed within the Austrian setting.

## Discussion

Within this study, we aimed to elaborate a concept for a potential evidence-based reimbursement process of DiHAs in Austria. For this, we developed a model to prioritise DiHAs directly relevant to the Austrian context (e.g., in accordance with the legal framework, available IT infrastructure, etc.). We further created a meta-directory of available DiHAs and applied a categorisation approach [11], in order to facilitate a structured overview of available DiHAs; finally, after applying the prioritisation criteria to the directory, we provide more detailed characteristics of prioritised DiHAs.

Although we identified numerous DiHAs from different registries (*n* = 181), only approximately one fifth (*n* = 38) was potentially relevant for implementation in Austria. The reason being that not all DiHAs were available in German language, and some DiHAs were categorised as having functions that did not fulfil the ASVG requirements for reimbursement as therapeutic aid or remedy (e.g., functions for documentation, information, prevention, prognosis or diagnosis). It should be noted, however, that the majority of the so far listed DiHAs in the German DiHA-directory [8] fall under the category of DiHA with therapeutic functions.

The indication areas of these prioritised DiHAs include mental disorders (*n* = 9), endocrine, nutritional and metabolic disorders (*n* = 7), cardiovascular disorders (*n* = 7), and "other" indications (*n* = 15). The majority of DiHAs report on CE marking (*n* = 29) and data protection information (*n* = 35). Yet, only a fragment of these DiHAs comply with or report transparently on, both regulatory and technology-specific requirements: Only ten (out of 38 prioritised DiHAs) report on risk class according to the MDR.

For the 38 prioritised DiHAs significant further barriers exist with regard to interoperability standards, with only 7 out of 38 being compatible with the current Austrian electronic health record standard [13,14]. The current available interoperability standard that is compatible with the Austrian health record is the HL7 CDA standard, with the transmission of so-called "CDA documents" as an interim solution. This technical difficulty could be overcome, if necessary, by the transmission of individual values using a new standard (the so-called "FHIR standard"). First demos with this newer standard are planned for the next 1–2 years [13,14]. There are ongoing discussions on whether the Austrian ELGA's technical compatibility could be a further filter criterion before evaluating DiHAs. However, since this creates a dependency on the respective development status of ELGA, it must be discussed whether compatibility with ELGA is essential.

When the 38 prioritised DiHAs are classified into the evidence levels of the NICE Evidence Standards assessment framework [15], it is clear that the prioritised DiHAs relevant to the ASVG are all classified in the highest evidence level C (DiHAs that function as interventions: guiding treatment, active monitoring and clinical calculations, providing or guiding a diagnosis, preventative behaviour change or allowing self-management of a diagnosed condition), with correspondingly high required evidence standards. Depending on further evidence categories within the evidence level C, these are described as high-quality observational or quasi-experimental studies as minimum evidence standard, and high-quality intervention studies (RCTs) as best practice standard. Perhaps in contrast to the minimum evidence standards described in the NICE assessment framework [15], requiring RCTs for all DiHAs may be proposed, as they are feasible and may combine classical methodological concepts [21] with new approaches of study conduct (e.g. routine data, remote trials).

Information on algorithms was not publicly available for any of the identified prioritised DiHAs, making assessments if (and to what extent) machine learning algorithms are in use impossible. Machine learning is an application of AI that provides systems the ability to automatically learn and improve from experience without being explicitly programmed. Machine learning focuses on the development of computer programs that can access data and use them to learn for themselves [15]. Due to potential continuous changes, new challenges when assessing machine learning technologies arise [15]. In a process where manufacturers apply for reimbursement, more information on algorithms used may be provided, as it is essential for assessing a DiHA thoroughly.

The development of DiHAs and their methodological evaluation is a highly dynamic field. For this reason, the AIHTA has performed and published two reports on DiHAs: diagnostic "symptom-checkers" [10], and tele medical diabetes applications [9]. Evidently, the former assessed DiHA would not have met the Austrian-relevant prioritisation criteria that were developed within this study. The DiHAs assessed in the latter report on telemedical diabetes programs meet the prioritisation criteria and two DiHAs are already embedded within regional diabetes disease management programs (DMPs) in Austria [9]. Suffice it to say that once a reimbursement process is implemented in Austria, established requirements (e.g., interoperability, data protection, evidence) need to be fulfilled also for these DiHAs.

Compliance is another important aspect to be considered when it comes to adopting DiHAs. In light of the aforementioned HTA report on telemedicine in the context of diabetes care [9], the compliance of one DiHA supporting an Austrian DMP was poor. That is, the drop out for this tele medically supported DMP was 41% (*n* = 522) and the non-compliance measured by how inactive participants were with regard to using the DiHA was estimated to between 20 and 30% (*n* = 1.169) based on two studies included within the HTA report, respectively [22,23]. Similarly, a real world data analysis on user engagement in the context of mental health apps found that only a small portion of users actually used the apps for a long period of time after installation [24].

Further, the successful use of digital technologies more broadly requires that participants have sufficient digital health literacy. The Austrian Health Literacy Survey 2020 [25] shows that the greatest challenge (compared to general health literacy) lies in the area of navigation skills (i.e., orientation in the health care system) and digital health literacy (i.e., dealing with digital technologies and resources). Hence, there may be a need to increase digital health literacy (for both patients and users) in parallel to reflecting on how and where DiHAs may be adopted.

In Austria, there are ongoing workshops between the AIHTA and the statutory health insurance and relevant stakeholders to discuss specific procedural and policy questions of implementing an evidence-based reimbursement process. In the thorough evaluation of DiHAs, the cooperation of different Austrian institutes with corresponding task distribution (e.g., assessment of data protection and interoperability) is necessary. In these workshops, a so-called "needs-based" approach was discussed within the identification phase: DiHAs could be developed based on specific needs of the population, rather than a "technology-based" approach that is, for instance, to be seen within the current German digital health fast track process [8].

On the EU-level, a taskforce with the aim of harmonising the evaluation of digital medical devices across the EU was initiated in 2021 by France and Germany [26]. Clinical evidence requirements under a harmonised taxonomy for DiHAs, while also taking into account the national implementation requirements, are currently being elaborated. Points of discussion, amongst others, are the timing of evidence generation, adequate study designs, appropriate handling of new study designs and real world evidence, and relevant comparator(s) and outcomes for DiHAs. Concepts like European Network for Health Technology Assessment (EUnetHTA) [27] could further be established to both improve efficiency and reduce redundant work when assessing DiHAs.

Our research article is best interpreted in light of its limitations. At present, there is no universal approach for the categorisation of DiHAs. While the approach by the iDiGA project [11] to classify available DiHAs was well suited for the creation of the DiHA meta-directory, it should be noted that numerous categorisation approaches exist [28]. However, since these are fairly similar, it is unlikely that the use of a different categorisation approach would have led to fundamentally different results. We did not calculate estimates for inter-rater reliability, such as Cohens Kappa, for the categorization of DiHA. Although this would have been more stringent, we are confident that the results have not been affected, as the applied categorisation approach [11] was stringent and qualitative consensus mechanisms were rarely needed when categorising available DiHAs. The expert consultations were not standardized in accordance with qualitative research methods for interview studies, and while this has inherent disadvantages, we believe that our approach was fit for purpose (the latter being gaining insights into technical knowledge [16], with information largely openly accessible and cited in this manuscript.

This study did not aim to assess the evidence for the described DiHAs or how these are best to be evaluated. However, we believe it is important to acknowledge a number of challenges, including optimal study designs and how to best incorporate the patient perspective within DiHA assessments. A recent analysis [29] assessed the risk of bias of trials on DiHAs adopted within the German fast-track reimbursement system and found that the great majority of trials had a high risk of bias, indicating need for improvement. There are promising novel study designs to be used for DiHA assessments, such as stepped-wedged randomised cluster trials. Evidence on the successful application of such designs is increasing [30]. With regard to the patient perspective, experience of approaches for patient input gained through the EUnetHTA [31] can be built on with a specific DiHA lens.

The conceptualised process presented in this paper is, amongst others, based on consultations of experts of the Austrian health system; as such, it is likely not directly transferrable to other health systems. For instance, a focus on DiHAs with therapeutic functions for chronically ill patients is proposed in the Austrian-relevant evidence-based reimbursement process designed in this study due to the Austrian legal framework (ASVG) [17]. However, for DiHAs with diagnostic or preventative functions targeting different user groups, a systematic approach will likely also be required in the future, similar to Germany [11].

Consequently, a number of questions would still need to be answered before this process can be refined and implemented. This may be considered a weakness for fast adoption. However, the concept was intended to depict an iterative process, showing options for decision makers and hopefully ensuring sustainable decisions on the specifics of a future reimbursement process that incorporates the perspectives of numerous stakeholders.

## Conclusion

The evidence-based reimbursement process designed in this study offers guidance for policy makers (e.g., using a prioritisation model to filter the high number of available DiHAs) in Austria and beyond, and may foster scientific debate regarding DiHA implementation. Further questions are yet to be answered before establishing a clear process of reimbursing DiHAs that incorporates regulatory, technology-specific as well as evidence-based criteria. Attention should be given to national implementation requirements (such as compatibility with electronic health records), criteria for re-assessment of DiHAs and remuneration schemes.

## Consent

NA

## Ethical approval

Not required.

## Funding

There was no external funding for conducting this research.

## Availability of data and materials

The datasets generated and/or analysed during the current study are not publicly accessible but can be made available from the corresponding author upon reasonable request.

## CRediT authorship contribution statement

**Gregor Goetz:** Conceptualization, Data curation, Formal analysis, Investigation, Methodology, Project administration, Visualization, Writing – original draft, Writing – review & editing. **Reinhard Jeindl:** Conceptualization, Data curation, Formal analysis, Investigation, Methodology, Project administration, Visualization, Writing – original draft, Writing – review & editing. **Dimitra Panteli:** Conceptualization, Supervision, Validation, Writing – original draft, Writing – review & editing. **Reinhard Busse:** Conceptualization, Supervision, Validation, Writing – review & editing. **Claudia Wild:** Conceptualization, Supervision, Validation, Writing – review & editing.

## Declaration of Competing Interest

The authors declare that the research was conducted in the absence of any commercial or financial relationships that could be construed asa potential conflict of interest.
